# A Study on the Relationship Between Learning Motivation and Learning Effectiveness of Personnel Based on Innovation Capability

**DOI:** 10.3389/fpsyg.2021.772904

**Published:** 2021-10-25

**Authors:** Guoqing Zhang, Chenin Chen

**Affiliations:** ^1^Business School, Shanxi Datong University, Datong, China; ^2^Graduate School of Business and Advanced Technology Management, Assumption University of Thailand, Bangkok, Thailand

**Keywords:** high-tech industry, innovation capability, learning motivation, learning effectiveness, structural equation modeling

## Abstract

Under the impact of intense competition in the face of globalization, the enhancement of the quality of human capital has become the primary goal for enterprises reinforcing the competitiveness as well as the power for constant growth and profit creation. It is the well-known norm of enterprises as well as the standard of human resource management; the enhancement of capability is the key activity of enterprises as well as the common task for modern people. Work and learning run parallel in order to cope with the rapid accumulation and change of knowledge; furthermore, in addition to enterprises providing opportunities for education, employees are requested to constantly update their training. Employees in the high-tech industry in Shanxi Province, as the research objects, were distributed 500 copies of one standard questionnaire, where 384 valid copies were retrieved, with a retrieval rate of 77%. The research results illustrate significantly positive effects of (1) learning motivation on innovation capability, (2) innovation capability on learning effectiveness, and (3) learning motivation on learning effectiveness. According to the results, providing the high-tech industry with more effective education curriculum planning and arrangement is expected.

## Introduction

Knowledge-based economy is, without a doubt, the most famous study in the twenty first century. Under the impact of intense competition in the face of globalization, the enhancement of the quality of human capital has become the primary goal for enterprises reinforcing the competitiveness as well as the power for constant growth and profit creation. It has become the well-known norm and the standard of human resource management for enterprises. Education, therefore, has become the emphasized task of enterprises. Merely the constant promotion of employees' productivity and enhancement of employees' professional skills could maintain employees' contribution to the organizations. Based on the investment in human capital, most enterprises would strongly prefer to invest in education curriculum and activity. However, it is speculated whether employees could have the best educational effectiveness in return for enterprises' investment in education, whether employees are willing to accept education when enterprises provide educational opportunity, and whether employees could directly apply the learning results in relation to work performance after receiving the education. These are the questions concerned by enterprises investing resources in educational opportunity and expecting to enhance competitiveness with improvement.

From previous employment experience, the decision is mainly based on educational background. This is exactly why citizens pursue higher education blindly. They do not value the actual ability to develop skills. Nowadays the working environment has changed into valuing employee's ability gradually (Dul and Ceylan, 2011; Rothes et al., 2017). The enhancement of capability has become the key activity of enterprises as well as the common task for modern people. To cope with the rapid accumulation and change of knowledge, work and learning run parallel; furthermore, enterprises, in addition to providing opportunities for education, request that employees engage in constant training. “Motivation” is the power of behavior, and learning is no exception. Indeed, successful learning activities promote strong motivation; learning activities without motivation tend not to convey the expected effect. Learning motivation is the most important driving force of learning behavior, as it facilitates learners to actively engage with the learning content. Moreover, it guides learners to grasp the learning direction as well as positively and continuously engage in learning activity to complete tasks and achieve the preset learning objectives. Past research pointed out learning motivation and learning behavior as key factors in learning effectiveness; education could induce learners' innovation capability and enhance educational effectiveness. When the correlations were discovered, it could provide effective assistance for human resource managers preceding education curriculum design, execution, and development (Brinkman, 2010; Brettel and Cleven, 2011). Therefore, there are a lot of discussions about training results (Wong and Wong, 2021). Many researchers can discuss the evaluation methods and affecting factors of training results from different perspectives (Prieto, 2012; Rothes et al., 2017). Besides, there are many discussions about creativity (Shao et al., 2019; Smadi and Raman, 2020). During the discussion of training results, learning motivation and learning behavior are often mentioned, however, the connection between employees' learning motivation, creativity, and training results is rare.

As a result, the relationship between learning motivation and learning effectiveness of personnel in the high-tech industry, based on innovation capability, is discussed in this study, expecting to provide the high-tech industry with more effective education curriculum planning and arrangement.

## Literature Review and Hypothesis

Aiming at adults' learning motivation and innovative performance, Cheng and Yi (2018) conducted empirical research and discovered remarkably positive relations between learning motivation and innovative performance. In the discussion of effects of learning climate and knowledge sharing on employees' knowledge transfer performance and innovative behavior, Nazir et al. (2018) carried out empirical research with learning motivation as the moderator and discovered notably positive effects of learning motivation on knowledge sharing and significantly positive relations between knowledge sharing and employees' innovative behavior. Sung et al. (2019) studied the correlations between students' learning motivation and creativity and discovered that learning motivation (intrinsic motivation and extrinsic motivation) could effectively predict creativity. The following hypothesis is therefore proposed in this study.

**H1**: Learning motivation reveals significantly positive effects on innovation capability.

Wang et al. (2018) revealed that engineering professionals with slightly high “innovation capability” would reinforce the “contextual performance.” In addition, Akram et al. (2018) pointed out innovation capability as the most critical factor in excellent innovation performance because of the characteristics of new products with a shorter life cycle and higher introduction in the market; therefore, employees in a company with better innovation capability would demonstrate higher work performance. Equally important, Mishra and Pandey (2019) considered that an individual with higher understanding and cognition of the environment and others could mutually deliver ample knowledge and information to further induce more innovation, enhance intelligent capital, and assist in work performance. In this case, the following hypothesis is proposed in this study.

**H2**: Innovation capability shows remarkably positive effects on learning effectiveness.

Similarly, Ho and Fu (2018) considered that effective connection of business personnel's core competencies and learning motivation could enhance education effectiveness, especially the relationship between “intrinsic motivation” and “results level.” Furthermore, Bednall et al. (2018) discussed the effects of the internal locus of control, self-efficacy, organizational commitment, and perceived interpersonal justice in remedial education on learning motivation and learning effectiveness and detected positive effects of learning motivation on educational reaction and learning performance. Roibu et al. (2019) studied the correlations among learning motivation, educational effectiveness, and work performance of employees in the high-tech industry and discovered that most employees in the high-tech industry participated in education due to intrinsic motivation, and behavior level in educational competency appeared to show better effectiveness. Accordingly, the following hypothesis is proposed in this study.

**H3**: Learning motivation presents notably positive effects on learning effectiveness.

## Methodology

### Operational Definition and Measurement of Variable

#### Learning Motivation

Referring to Qi et al. (2019), the conceptual structure of participation in learning and dropping out based on the introduction of human resource development are revised for this study. The operational definitions for adults' learning motivation are explained below.

1. Career progression: It could benefit career development, the enhancement of functional competency for work, and promotion.
2. Social relations: Making friends and expanding interpersonal relations and social network.
3. External expectation: Participating in learning activity due to the requirement and expectation of supervisors or teachers in order to conform to others' requirements, obeying the request and encouragement of supervisors or teachers, and being influenced by others' participation in education.
4. Fun to seek knowledge: To enhance professional growth and cognitive interest as well as broaden horizons with learning.
5. Self-fulfillment: Pursuing personal development to enhance adaptation and self-understanding.

#### Innovation Capability

Referring to Jia et al. (2019), individual innovative behavior is divided into innovative idea generation and innovative idea execution in this study.

1. Innovative idea generation: Members perceive problems and come up with solutions.
2. Innovative idea execution: Members seek for supporters that agree with the new ideas and attempt to establish supporter alliance for the ideas and eventually build an innovative model with such ideas.

#### Learning Effectiveness

Referring to Le and Lei (2019), learning outcome is divided into cognitive outcome, skill-based outcome, and emotional outcome in this study.

1. Cognitive outcome: Cognitive outcome is decided by trainees' familiarity with the principles, facts, skills, programs, or processes emphasized in the education curriculum to measure the knowledge learned in the education curriculum. Traditionally, the cognitive outcome is evaluated with a paper-and-pencil test.
2. Skill-based outcome: Skill-based outcome is used for evaluating state-of-the-art, motor skills, or behavioral outcome, including skill acquisition or learning; and, the application of such skills to work (learning transfer) is mostly measured with field observation.
3. Emotional outcome: Emotional outcome contains attitude and motivation, as the measurement aiming at trainees' responses to and satisfaction with educational planning, educational equipment, and educational content to understand the factors in the success of education and the obstacles to education. A survey is generally applied to collect relevant information for evaluating trainees' emotional outcome.

### Research Object

Employees in the high-tech industry in Shanxi Province are selected as the research objects. A total of 500 copies of one standard questionnaire are distributed and 384 valid copies are retrieved, with a retrieval rate of 77%.

### Research Method

Structural equation modeling (SEM) is used for testing the research structure in this study. Structural equation modeling is divided into a confirmative factor analysis (CFA) model, which is also called the measurement model in structural equation modeling, to connect manifest variables with latent variables, and a structural model (also named latent variable modeling), which is mainly established among latent variables and is similar to path analysis. The difference lies in manifest variables being used for path analysis, but latent variables for the structural model.

The structural model is complementary to the measurement model; the structural model requires the measurement model for considering variable measurement error, while the measurement model requires the structural model for understanding the cause-and-effect relationship among latent variables. Structural equation modeling achieves the mutual needs of both models and simultaneously covers the measurement model and structural model, allowing measurement error in variables, as in the measurement model, allowing error (or residual) in equations, and estimating the cause-and-effect relationship among latent variables, as in the structural model.

The model fit can be evaluated from preliminary fit criteria, overall model fit, and fit of internal structure of model.

### Test of Reliability and Validity Analysis

The reliability of dimensions in this study reaches 0.7, revealing high reliability of such dimensions. The construct validity of the scale in this study is analyzed with confirmative factor analysis. Table 1 shows good convergent validity and construct validity of the scale in this study. The standardized regression coefficients of indicators of latent dimensions achieve significance, between 0.5 and 0.95, and the measurement error does not appear negative meaning that it is acceptable.

**Table 1:** Overall linear structural model analysis result.

**Evaluation item**	**Parameter/evaluation standard**	**Result**
Internal fit	Learning motivation → innovation capability	0.32^**^
	Innovation capability → learning effectiveness	0.29^**^
	Learning motivation → learning effectiveness	0.37^**^

** *p < 0.01*.

## Analysis Result

### Correlation Analysis

The correlation analysis results present remarkable correlations among learning motivation, innovation capability, and learning effectiveness. Such results reveal the possibility of multicollinearity among research dimensions. Nested model analysis could be used for solving the problem. The notable correlations among research dimensions confirm the research hypotheses.

### Overall Model Discussion

In terms of overall model fit, the overall model fit standards χ^2^/df = 1.587, smaller than the standard 3, and RMR = 0.006 reveal the proper results of χ^2^/df and RMR. Furthermore, the chi-square value is sensitive to sample size so it is not suitable for directly judging the fit. However, the overall model fit standards GFI = 0.974 and AGFI = 0.938 are higher than the standard 0.9 (the closer GFI and AGFI are to 1, the better the model fit) so the model presents better goodness-of-fit indices.

### Research Hypothesis Discussion

The structural equation modeling testing results (Table 1) show the effects of “learning motivation” and “innovation capability” on “learning effectiveness.” Aiming at the above objectives, the influence is explained.

1. The direct and positive effect of “learning motivation” → “innovation capability” is 0.32^**^, so H1 is supported.
2. The direct and positive effect of “innovation capability” → “learning effectiveness” is 0.29^**^, so H2 is supported.
3. The direct and positive effect of “learning motivation” → “learning effectiveness” is 0.37^**^, so H3 is supported.

As we used the nested model to test hypotheses, the chi-square test is used because each nested model presents the difference of a degree of freedom; in this case, when the difference between the chi-square value of the nested model and the chi-square value achieves significance, the setting of path coefficient = 0 is significant. The research results reveal the significance of the model. The nested model analysis results are shown in Table 2.

**Table 2:** Nested model analysis.

**Model**	**χ^2^**	**Δχ^2^**	**GFI**	**CFI**	**RMSEA**
Theoretical model	241.37		0.974	0.962	0.04
Model 1: Hypothesis test	244.63	3.26^*^	0.974	0.962	0.04
Model 2: Hypothesis test	248.74	4.11^*^	0.974	0.962	0.04
Model 3: Hypothesis test	253.57	4.83^*^	0.974	0.962	0.04

* *p < 0.05*.

## Discussion

In the structural composition analysis of employees in the high-tech industry, educational courses aim to impart management-related knowledge to employees in the field and cultivate their leadership ability. The curriculum design stresses on the combination of theory and practice so that employees in the high-tech industry, after learning, can apply the learned competencies to practical work. For this reason, employees in the high-tech industry are educated to actively and positively engage in relevant courses and clarify the learned competencies to promote work quality and work efficiency and effectiveness. As a result, employees in the high-tech industry should modify their learning attitude, create learning strategies, cultivate good learning habits, and apply learned knowledge and skills to the workplace for generating and implementing innovative ideas to construct personal high learning effectiveness. Education institutions in the high-tech industry could revise the education curriculum, to talent education and other on-the-job training, based on innovation capability to reinforce learning motivation, facilitate positive learning behavior, and further induce innovation capability to promote learning effectiveness. In order to strengthen the learning behavior of employees in the high-tech industry, the innovation capability should be reinforced to further enhance learning effectiveness. Such innovations rely on the establishment of a counseling mechanism to guide systematic learning and learning situations for enhancing learning performance and learning effectiveness. Blamiresa and Peterso (2014) believed that junior staff and middle managers are the foundations in a department. Therefore, it is necessary to reinforce their learning behavior and creativity to improve their training results. Han et al. (2013) thought this depended on establishing a counseling system. It can guide them to learn systematically and form a suitable learning environment to enhance training grades and results (Kuoa et al., 2017).

## Conclusion

The research findings show that innovation capability is the major factor in the learning motivation of employees in the high-tech industry that enhances learning effectiveness, where innovation capability is the best measurement variable of learning effectiveness. Innovation capability originates from the change in learning behavior that good learning attitude, effective learning strategy, and cultivation of good learning habits can induce individual innovation capability and enhance personal learning effectiveness. The motivation of employees in the high-tech industry to participate in the talent education curriculum relies on interest in pursuing knowledge, career progression, and self-fulfillment. For this reason, talent education institutions, when designing and planning an education curriculum, should take employees' interests and needs into account and understand their interests and needs through surveys or discussions. In order to secure a higher rate of participation in an education curriculum, taking such factors into account and incorporating them into the education curriculum would be the most effective way. Consequently, the reinforcement of learners' innovation capability in the curriculum design is the most effective way to enhance learning effectiveness. How to have employees in the high-tech industry apply the learned management theory to practical work with the combination of theory and practice should be a major task for those concerned with planning talent education curriculum design. The practicable methods contain personal project reports of employees in the high-tech industry, i.e., applying management theories, concepts, tools, and tactics learned in the training to the current work, or an action plan checklist, i.e., applying the learned management theories, concepts, tools, and tactics to improve work performance, and setting goals with execution to prove what has been learned. Shriki (2013) indicated that employees working in the high-tech industry can improve their training results through creativity, and creativity is an important impact factor to improve their learning motivation and training results (Lee and Yang, 2015). These research results should be widely promoted and applied. Therefore, the high-tech industry should also develop educational training courses while doing personnel training. This would be helpful to reinforce the learning motivation of employees in the high-tech industry and promote positive learning behaviors. It can also inspire creativity to enhance training results.

## Data Availability Statement

The original contributions presented in the study are included in the article/supplementary material, further inquiries can be directed to the corresponding author.

## Ethics Statement

The present study was conducted in accordance with the recommendations of the Ethics Committee of Shanxi Datong University. Written informed consent was obtained from all the participants.

## Author Contributions

GZ and CC revised and approved the submitted version of the manuscript. GZ performed the initial analyses and wrote the manuscript. CC assisted in the data analysis. All authors contributed to the article and approved the submitted version.

## Funding

The authors appreciate financial support for the research and publication of this article (2020w105) from the Department of Education, Shanxi Province, China.

## Conflict of Interest

The authors declare that the research was conducted in the absence of any commercial or financial relationships that could be construed as a potential conflict of interest.

## Publisher's Note

All claims expressed in this article are solely those of the authors and do not necessarily represent those of their affiliated organizations, or those of the publisher, the editors and the reviewers. Any product that may be evaluated in this article, or claim that may be made by its manufacturer, is not guaranteed or endorsed by the publisher.
